# Supplementary material for: Cognitive job crafting as mediator between behavioral job crafting and quality of care in residential homes for the elderly
Source: PLoS One. 2020 Dec 15;15(12):e0243726. doi: 10.1371/journal.pone.0243726 (PMC7737976; doi:10.1371/journal.pone.0243726)
Supplement: Attachment — Submitted filename: Responses to reviewers.docx [file pone.0243726.s003.docx]

The manuscript has improved, but the authors need to address the issues raised by both reviewers. In addition to the comments of the reviewers (at the bottom of this letter), I would like the authors to address the following points concerning their work:

1. The authors have not yet adequately explained why cognitive crafting would act as a mediator between the more behavioral aspects of crafting and quality of care.  Could cognitive crafting precede the more behavioral aspects of crafting?

Recent studies suggest that behavioral crafting (task and relational) can cause changes at the cognitive level (Unsworth, Mason, & Jones, 2004; Zhang & Parker, 2018). In this sense, Unsworth et al. (2004) pointed out that “*group members cognitively frame their comparisons such that they compare themselves with other groups on dimensions that represent in-group strengths (…). For example, members of a highly productive group would tend to evaluate groups in terms of their productivity, but a less productive group might choose to focus on other attributes, such as friendliness*” (p.5). *In our research, following this suggestion, we consider that employees could cognitive reframe the meaning of their jobs by means of the tasks (Task crafting) they develop and the relationships they have at work (Relational crafting)* (Theoretical framework and hypotheses section)

Various studies have linked job crafting with performance, including the quality of service offered by employees (Lichtehthaler and Fischbach, 2019; Rofcanin, Bakker, Berber, Gölgeci and las Heras, 2018; Rudolph et al., 2017; Tims et al., 2015; Yepes-Baldó et al., 2018). Compared to previous studies, the main novelty of our proposal lies in the fact that we analyze job crafting as a dynamic process and differentiate between behavioral job crafting (tasks and relational crafting), as an antecedent, and cognitive job crafting as a consequence, and as a mediator between it and the quality of care .The results of previous studies [9–11] reveal different relationship dynamics for task and relational job crafting, on the one hand, and cognitive job crafting, on the other.

2. The authors have not yet answered the question of a previous reviewer.  For the analyses, why were the models tested as separated mediation models using PROCESS? As noted by a previous reviewer,  the data would be more parsimoniously handled with SEM models, where they could a) include all variables simultaneously, and b) account for attenuation caused by measurement error when you model with latent variables, and c) test competing positions of the variables so that you could better establish which one is better position as a mediator, predictor, etc. So, the authors should address this issue.

Hayes (2013) showed the similarity in results between PROCESS and an SEM program. Additionally, PROCESS estimates each equation included in a model to be tested separately (Hayes, Montoya, & Rockwood, 2017).

Secondly, as pointed out by Hayes et al. (2017), SEM is more suitable with large samples, as it relays on large sample asymptotic theory. For this reason, as we have a sample of 226 participants, we decided to use PROCESS.

Related to latent variables, we reduced them to observed variable proxies (averages of indicators), which make them, by definition, observed and not latent (Hayes et al, 2017).

In relation to measurement error, any analysis that can be expressed in the form of a linear regression model is not exempt of it, including SEM. On the other hand, latent variable mediation analysis may be more accurate in the estimation of effects than observed variable analysis, but less powerful in detecting them (Hayes et al, 2017).

A limitation of PROCESS is that only one exogenous variable can be entered in a single analysis. That is the reason why we used 2 separate mediation models. Additionally, as we aforementioned, previous research has arisen that the 3 components of job crafting have different relationship dynamics for task and relational job crafting, on the one hand, and cognitive job crafting, on the other. So, our decision is based on theoretical and empirical reasons.

3. What are the practical implications for the people involved in their research? How should they change the way they work based on the results of the research? We need to know the implications of the research for work practices.

Following a psychosocial perspective, we considered important to point out the context key aspects for human resource management. In order to allow people “to change the way their work” we focused on the organizational context and managerial practices. We consider that it is important to guarantee the working conditions that allow employees turn the job they have into the job they want (Wrzesniewski , Berg, and Dutton, 2010).

We added this consideration in the text (Implications for theory and research).

==============================

**Comments to the Author**

**Reviewer #2:**

Thank you for your revisisions and addressing my previous comments. These are mostly addressed, and I have a few more clarification points:

a) The inclusion of the Wrzesniewski and Dutton definition should be integrated into one of the paragraphs and not be a standalone sentence.

Done. We integrated it with the subsequent paragraph (Theoretical framework and hypotheses section)

b) I don’t feel the second original point on why do these relationships happen (from line 80 onwards) is evident. The revisions are still descriptive, although the work of Ashforth and Kreiner helps with this. But this could still be unpacked further, and especially in explaining how and why job crafting is associated with quality of care.

Recent studies suggest that behavioral crafting (task and relational) can cause changes at the cognitive level (Unsworth, Mason, & Jones, 2004; Zhang & Parker, 2018). In order to explain better this relationship, we added the following in the text (Theoretical framework and hypotheses section):

*“group members cognitively frame their comparisons such that they compare themselves with other groups on dimensions that represent in-group strengths (e.g., Hogg, Abrams, Otten & Hinkle, 2004). For example, members of a highly productive group would tend to evaluate groups in terms of their productivity, but a less productive group might choose to focus on other attributes, such as friendliness” (p.5). In our research, following this suggestion, we consider that employees could cognitive reframe the meaning of their jobs by means of the tasks (Task crafting) they develop and the relationships they have at work (Relational crafting).*

Secondly, various studies have linked job crafting with performance, including the quality of service offered by employees (Lichtehthaler and Fischbach, 2019; Rofcanin, Bakker, Berber, Gölgeci and las Heras, 2018; Rudolph et al., 2017; Tims et al., 2015; Yepes-Baldó et al., 2018). We added some additional explanations in Theoretical framework and hypotheses section:

*Compared to previous studies, the main novelty of our proposal lies in the fact that we analyze job crafting as a dynamic process and differentiate between behavioral job crafting (tasks and relational crafting), as an antecedent, and cognitive job crafting as a consequence, and as a mediator between it and the quality of care.*

*The results of previous studies [9–11] reveal different relationship dynamics for task and relational job crafting, on the one hand, and cognitive job crafting, on the other.*

c) In terms of the Teoh et al. paper, the intention was not so much to summarise the findings of it, as actually the description and links between psychosocial working conditions and quality of care is not directly relevant. Instead, that paper highlights how quality of care can be operationsed in different ways and that it rather complex. What is needed here is for the paper to consider what is quality of care and how it is measured here relates to performance more generally and/or other measures of quality of care. The revised new paragraph (Line 131, During the past decades…) does a decent job at this, but it needs to better link in with the next paragraph on the review. This paper does highlight the review’s call for longitudinal and mediating research, which this study actually answers, and it would be good to use this as additional justification to strengthen the need for this study.

In order to clarify all these points, we included the following paragraph in Theoretical framework and hypotheses section:

*Following these suggestions and taking into account that job crafting is a modification of working conditions initiated by the employees, the present research propose job crafting as an antecedent of quality of care and a longitudinal design. Secondly, we propose cognitive crafting as a mediator between behavioral crafting (tasks and relationships), which modify working conditions, and quality of care.*

d) With regards to the point “As we used a 5-point Likert scale, we consider 3 or higher a medium to high score. We indicated this in the text”, I don’t think this is needed. What is the justification of the 3 and above as a medium to high score? Is this a cut-off determined as part of the questionnaire or something that was decided here? If there is no set comparison (normed) score than its not appropriate to say its high.

This is not a cut-off determinate by the questionnaire. The scale used is a 5-point Likert scale where 1 was “hardly ever”, and 5, “very often” (for job crafting scale) or 1, “very seldom or never”, and 5, “very often or always” (for quality of care). In order to avoid possible misunderstandings, we avoid the term medium-high and explained better the meaning of the results (Results-Descriptive statistics section):

*In all cases, the scores for job crafting and quality of care variables were above 3 in a 5-points scale. Following the scale range these results indicated that employees sometimes job craft, and often considered that quality of care in their workplaces was appropriate.*

e) There are elements of duplication that can be removed from this paper. For example there is no need to describe the five-point QoC scale in the Introduction and then again in the Method section. Equally if Cronbach Alpha scores are available in the Table 2 and it is mentioned that they all have good reliability then there is no need to mention them also in the measures section.

In relation with Westerberg and Tafvelin instrument, in the Introduction we justified it use while in Materials we described the instrument.

Related to alphas, in Materials section we described results obtained in the original researches, avoiding the present research results, while in Table 2 we included our results in the present research.

f) For H1 and H2, was it hierarchical multiple regressions that were carried out or simple linear regressions? If it is the former, which it should be, then this should be made clearer and the control variables also acknowledged.

We clarified in the text (Test of Hypotheses section) that we used hierarchical multiple regression, introducing control variables as step 1 and the different independent variables as step 2. As indicated in the text, *No significant effect of the control variables (gender, age, permanent contract, managerial position, night shift) was found*.

g) Cognitive job crafting T2 should be made clearer within the hypotheses. While ideally there would have been three time points (but two is really good as well – especially as it’s a year apart), as this is not the case here there should be some consideration as to why cognitive job crafting at Time 2 was used and not that from Time 1.

We added T2 more clearly within the hypothesis. We used cognitive crafting at T2 to test if changes in behavioral crafting could affect the cognitive reframing. If we would use cognitive crafting T1 we couldn’t assure the effect of behavioral crafting on cognitive crafting as they were measured simultaneously.

h) Page 4 line 66: “the healthcare sector has been characterized by reducing and slow growth of costs” – this sentence isn’t clear, is it reducing or is it having slow growth?

We agree. We have deleted “reducing and” as the healthcare sector has been characterized by slow growth of costs (Introduction section).

**Reviewer #3:**

I consider this to be a very interesting job as it applies job crafting to a group of professionals who were traditionally analyzed from the wrong perspectives.

The design of the work is very appropriate as it combines the cross-cultural perspective with an exhaustive control over the variables of the study.

I have a question regarding the tests they have used:

a. In which languages have the work questionnaires been applied? Spanish and Swedish? (page 10)

Yes, all questionnaires were administered in their Spanish and Swedish versions, as indicated in Materials section.

b. Did they apply the questionnaires in "paper and pencil" format? (page 9)

Yes, we applied the questionnaires in a paper and pencil format. We added this information in Participants and procedure section.

c. With an "online" application of the questionnaires could they have achieved greater success in answering? (page 11)

Participant had time during working hours to answer the questionnaire. With an online application the difficult would be to have access to enough electronic systems to answer and, on the other hand, it is more difficult to control who is answering.
